# Vancomycin nephrotoxicity: Vancomycin tubular casts with characteristic electron microscopic findings 

**DOI:** 10.5414/CNCS109817

**Published:** 2019-12-12

**Authors:** Ngoentra Tantranont, Chizoba Obi, Yosu Luque, Luan D. Truong

**Affiliations:** 1 Department of Pathology, The Houston Methodist Hospital and Weill Cornell Medical College, Houston, TX, USA,; 2 Department of Pathology, Faculty of Medicine Siriraj Hospital, Mahidol University, Bangkok, Thailand,; 3 Department of Medicine, Renal Section, The Houston Methodist Hospital, Houston, TX, USA, and; 4 Department of Nephrology and Transplantation, Tenon Hospital, Paris, France

**Keywords:** vancomycin, nephrotoxicity, tubular casts, immunostain, electron microscopy, biopsy

## Abstract

We herein report a 46-year-old man with diabetes who developed acute kidney injury and oliguria after receiving vancomycin to treat his foot infection. Renal biopsy revealed typical features of advanced diabetic nephropathy as well as features of acute vancomycin nephrotoxicity. Several changes typical for acute vancomycin nephrotoxicity, but hitherto not adequately described, were seen. There was an element of acute tubulointerstitial injury associated with frequent tubular casts consisting of typical hyaline casts, pale glassy material suggestive of uromodulin, and distinctive features suggestive of vancomycin deposition. Coprecipitation of vancomycin and uromodulin was confirmed by immunostain. Electron microscopic study showed features supportive for the diagnosis of diabetic nephropathy and distinctive concentric appearance of vancomycin tubular casts within the fibrillary background of uromodulin. The patient’s renal function improved rapidly after cessation of vancomycin and initiation of steroid therapy, suggesting that vancomycin-associated tubular injury is potentially reversible over time with proper management.

## Introduction 

*Staphylococcus aureus* infection is often successfully controlled by methicillin or penicillin. 

However, the occurrence of methicillin-resistant *Staphylococcus aureus* infection is increasing worldwide. Vancomycin is the drug of choice in this situation and increased use has led to increased frequency of vancomycin-related renal complication. The incidence of vancomycin-related nephrotoxicity ranges from 12 to 43% [1, 2]. Many risk factors are known, including vancomycin concentration of > 20 mg/mL, admission to critically ill, ICU patients, higher cumulative number of organ failures, and cirrhosis [1, 3]. Currently, the mechanism of vancomycin nephrotoxicity is not well established. 

We wish to report a case of vancomycin nephrotoxicity and describe the renal biopsy in this condition including the novel electron microscopic findings. Previously reported cases of biopsy-documented vancomycin nephrotoxicity are also reviewed. 

## Case description 

A 46-year-old man with poorly controlled diabetes was admitted with a left great toe wound with serous drainage and progressive swelling. Past medical history included hypertension, diabetes, and hyperlipidemia. Home medication included metformin, lisinopril, and lovastatin, last taken 2 weeks prior to this admission. Physical examination showed a well-developed man, with a blood pressure of 154/98 mmHg, normal temperature, left foot and leg covered with dressing. His serum creatine before this hospitalization was normal at ~ 0.9 mg/dL. Laboratory studies at admission showed a serum creatinine of 1.0 mg/dL, blood glucose of 408 mg/dL, and HbA1c of 16.6 mg/dL. Serum electrolytes and liver function tests were normal. Additional studies during hospitalization to evaluate the acute kidney injury included a urine protein excretion of 950 mg/day without urine eosinophil and normal serologic studies (antinuclear antibody, rheumatoid factor, and complement levels). A normal renal ultrasound MRI showed left first toe with enhancing edematous changes at the distal phalanx. Insulin, piperacillin/tazobactam (3.4 g every 6 hours), and vancomycin (1 g every 8 hours) were started at day 1 of admission. Wound culture grew methicillin-resistant staphylococcus. Left big toe amputation was done at post-admission day 4. There was no perioperative hemodynamic instability. Vancomycin trough levels were 17.5 mg/L at day 1 and 29.1 mg/L at day 5. Serum creatinine progressively increased from 0.8 mg/dL at day 1 to 1.9 mg/dL at day 5, with no associated change in urine output (1,000 – 1,800 mL per 24 hours, respectively). Oliguria and volume overload developed at day 6, with serum creatinine increasing progressively to a peak of 7.6 mg/dL at day 12. Vancomycin and lisinopril were discontinued at day 7. Renal biopsy was done on day 13. After the renal biopsy diagnosis, steroid was started at day 15 (intravenous solumedrol 250 mg/daily for 2 days, followed by oral prednisone 40 mg/day for 2 weeks, and then tapered by 20 mg every 2 weeks). The patient was discharged on day 19, at which time serum creatine was 3.9 mg/dL. At most recent follow-up at day 75, serum creatine was 3.1 mg/dL. The patient was lost for long-term follow-up. 

## Renal biopsy findings 

**Figure 1.**
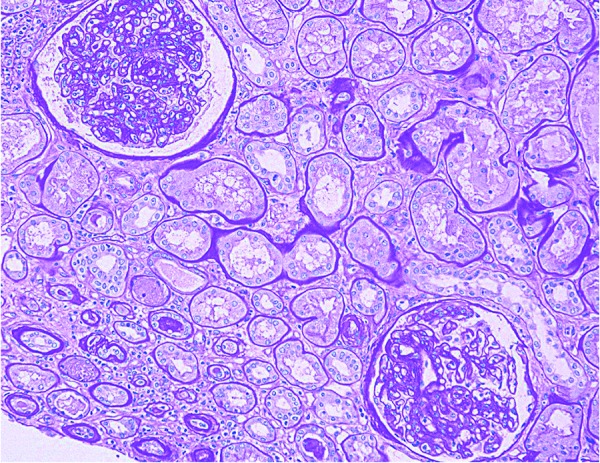
Diabetic nephropathy is noted including mesangial sclerosis, thickened glomerular capillary wall, and thickened tubular basement membrane (Periodic acid-Schiff, × 200).

**Figure 2.**
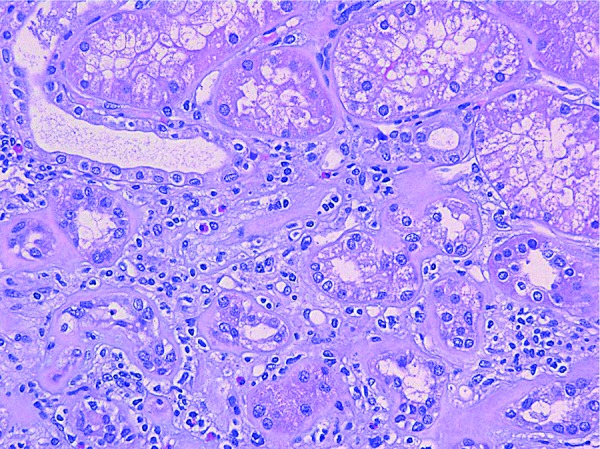
Tubulointerstitial nephritis including tubular atrophy, interstitial fibrosis, and interstitial inflammation with few eosinophils (H & E, × 200).

**Figure 3.**
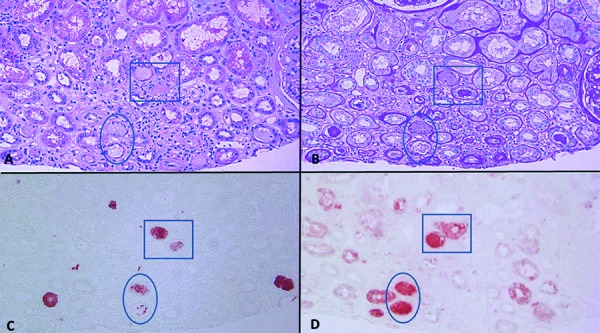
Tubular casts of different appearance are noted on consecutive tissue sections (A and B; (square and circle)), displaying partial overlapped staining for vancomycin (C) and uromodulin (D) (H & E for A, periodic acid-Schiff for B, and immunostain for C and D; × 100 for all panels).

**Figure 4.**
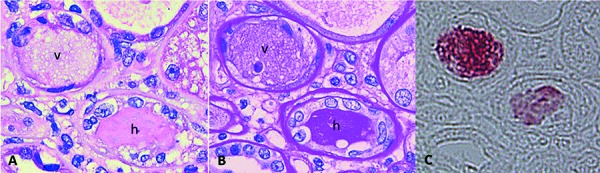
A and B: A tubular cast (v) with changes characteristic for vancomycin deposition: packed aggregation of spherules with central clearing, imparting a “bubble” appearance. Another tubular cast with a hyaline appearance (h). C: Both types of casts are stained positive for vancomycin (H & E for A, periodic acid-Schiff for B, immunostain for C; × 400 for all panels).

**Figure 5.**
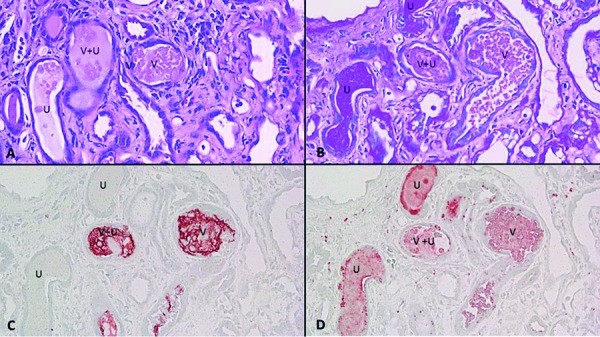
Consecutive tissue sections (H & E in A, periodic acid-Schiff in B, vancomycin immunostain in C, and uromodulin immunostain in D) show different types of tubular casts. Type V seems to represent pure vancomycin cast characterized by aggregated or individual eosinophilic globules, stained strongly for vancomycin, but also weakly for uromodulin. Type V+U seems to display features of both vancomycin deposit against a background of uromodulin, which is confirmed by immunostain. Type U seems to represent pure uromodulin cast, which is positive in PAS and uromodulin immunostain, but negative for vancomycin (× 200 for all panels).

**Figure 6.**
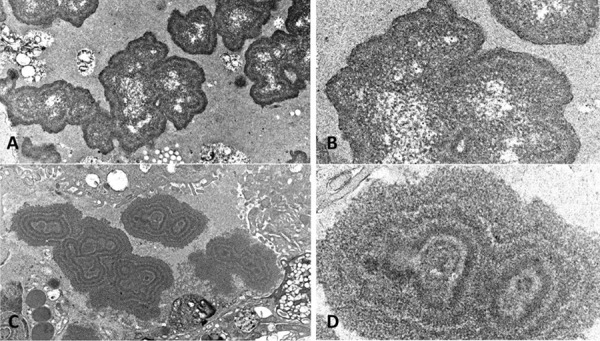
Vancomycin tubular casts display distinctive ultrastructural features: electron-dense or variegated granular material forming concentric laminar globular structures that are isolated or form aggregates. These structures maintain an amorphous core (A and B) or are entirely crystalized (C and D). These casts are set against a background of pale granular or fibrillary material suggestive of uromodulin (electron microscopy; × 12,000 for A and C, × 25,000 for B and D).

The renal biopsy was submitted to routine light microscopic, immunofluorescent, and electron microscopic studies. It was also submitted to immunostain for Mib-1 (a marker for cell division), myoglobin, vancomycin, and uromodulin. Light microscopic findings included diffuse mesangial matrix expansion with some nodular sclerotic lesions (Figure 1). Few segmentally sclerotic glomeruli were identified. There was chronic tubulointerstitial injury characterized by tubular atrophy and interstitial fibrosis, involving ~ 50% of cortical tissue area (Figure 2). The interstitium was focally infiltrated by lymphocytes, plasma cells, some eosinophils and neutrophils (Figure 2). There was diffuse tubular basement membrane thickening (Figure 1). Many clusters of tubules displayed reactive epithelial changes with sloughing of cells into lumen and dystrophic calcification. Tubular casts were frequent and displayed various morphologies (Figure 3). Some appeared as typical hyaline casts or casts with features suggestive of uromodulin accumulation (Figures 4, 5). Some tubular casts displayed distinctive features suggestive of vancomycin deposition as packed clusters of spherules with central clearing, imparting a “bubble” appearance (Figure 4), or ill-defined or nodular collections of pale eosinophilic material, which were isolated or formed contiguous aggregates (Figure 5). A background of pale glassy material characteristic of uromodulin was noted in some of these casts, suggesting vancomycin/uromodulin coprecipitation (Figures 4, 5). Structures suggestive of necrotic cells were also noted in some of these casts. The arteries showed severe intimal fibrosis, and many arterioles showed severe hyalinosis. Immunostain for uromodulin or vancomycin confirmed the presence of these molecules in different tubular casts. In addition, their coprecipitation was also noted in some of these tubular casts (Figures 3, 4, 5). Mib-1 immunostain revealed multifocal staining of tubular cell nuclei supporting the presence of acute tubular injury. Immunostain for myoglobin was negative. Immunofluorescent study showed no staining for immunoglobulins or complement components for any renal compartment. Electron microscopic study showed diabetic glomerulosclerosis including diffuse thickening of the lamina densa of the glomerular basement membrane and mesangial matrix expansion corresponding to the light microscopic findings. Vancomycin tubular casts displayed distinctive ultrastructural features as electron-dense or variegated granular material forming concentric laminar globular structures consistent with vancomycin aggregates. These structures maintained an amorphous core (Figure 6A, B) or were entirely crystalized (Figures 6C, D). These casts were set against a background of pale granular or fibrillary material suggestive of uromodulin. 

The renal biopsy diagnoses included changes consistent with acute vancomycin nephrotoxicity (acute tubular necrosis, acute interstitial nephritis, and vancomycin tubular casts) against a background of advanced diabetic nephropathy and severe arterial nephrosclerosis. 

## Discussion 

Up to the present, there have been 19 individual case reports of vancomycin-related nephrotoxicity, with renal biopsy results summarized in Table 1. Seven showed acute tubular necrosis (ATN), 8 showed acute tubulointerstitial nephritis (TIN), 2 showed acute TIN with predominant ATN features, and 2 showed granulomatous TIN. The morphologic changes in cases with ATN were consistent with nonspecific nephrotoxic type, which are necrosis of tubular cells, predominantly proximal tubules, and mild interstitial inflammation. One case showed allergic features that were consistent with drug rash with eosinophilia and systemic symptoms (DRESS). All 4 cases that received piperacillin/tazobactam developed features of ATN; 2 isolated ATN and 2 acute TIN with ATN. In most of the cases with acute TIN, the changes were consistent with nonspecific allergic type, as there was significant interstitial inflammation with eosinophils. Three cases had systemic allergic symptoms (2 DRESS and 1 rash). One out of 2 cases of granulomatous TIN was associated with rash. According to a report of 9 cases by Luque et al. [2], vancomycin casts were first described as granular proteinaceous casts appearing as non-crystalline spherical formations, 100 – 900 nm in size by scanning electron microscopy. The casts were confirmed by immunohistochemistry, infrared spectroscopy, and electron microscopy with immunogold labeling. In addition, the vancomycin casts are entangled with uromodulin indicating the obstructive nature of vancomycin-associated casts. 

In our case, the patient developed acute TIN with predominant ATN, which was most probably due to vancomycin nephrotoxicity. The development of nonoliguric acute kidney injury subsequently progressing to oliguric acute kidney injury with volume overload argues against volume depletion as a cause of acute kidney injury. The interstitial infiltrate is composed of mixed inflammatory cells with eosinophils, which are consistent with nonspecific allergic type TIN. The renal biopsy in the current case helps illustrate several novel and distinctive changes that may be of both diagnostic and pathogenetic significance. There are tubular casts with characteristic features that may imply vancomycin deposition with or without associated uromodulin. These findings are indeed confirmed by immunostain for vancomycin and uromodulin done for the same casts on consecutive tissue sections. In addition, these vancomycin casts display characteristic ultrastructural features revealed by transmission electron microscopy: electron-dense or variegated concentric laminated structures. These are seen against a fibrillary background, consistent with uromodulin fibrils, in keeping with the immunohistochemical findings. These findings expand the ultrastructural morphologic spectrum of vancomycin casts as originally depicted by scanning electron microscopy [2]. Indeed, tubular casts with the same characteristic ultrastructural appearance have been noted in most of the other renal biopsies with features of vancomycin nephrotoxicity that we have encountered. The significance of vancomycin deposition in tubular lumens is not clear. This finding raises the possibility of a direct tubulotoxicity of vancomycin. Alternatively, this accumulation may be of a secondary nature and may merely reflect an unrelated pre-existing chronic/acute tubulointerstitial injury that led to a failure to clear a therapeutically acceptable renal load of vancomycin. Although the pathogenesis remains unclear, a coprecipitation of vancomycin and uromodulin suggests that vancomycin accumulation may be facilitated at least at the single nephron level by urine obstruction and the in situ presence of uromodulin. 

Patients treated with vancomycin are often affected by other conditions or treated with other medications that by themselves can cause acute kidney injury. Confirming vancomycin nephrotoxicity would be instrumental for management, since aside from cessation of vancomycin, treatment may include steroids, leading to a favorable outcome noted in the current case and in several previous reports. The current case also helps illustrate the diagnostic utility of renal biopsy, which shows characteristic findings including typical vancomycin tubular casts and the coexistence of acute tubular necrosis and acute interstitial nephritis. The unique ultrastructural features of vancomycin tubular casts are also demonstrated. 

## Funding 

The authors received no specific funding for this work. 

## Conflict of interest 

All authors have no conflict of interest to declare. 

**Table 1.** Previous case reports of vancomycin-related nephrotoxicity that had renal biopsies.

	Author, year	Age, gender	Indication for therapy	Other disease	Other nephrotoxins	LM findings
1	Codding et al. 1989 [4]		Endocarditis	Rash	Not known	Granulomatous TIN
2	Michail et al. 1998 [5]		Chest infection		Not known	Acute TIN
3	Wai et al. 1998 [6]	64, M	Wound infection, endocarditis	DRESS syndrome		Acute TIN
4	Hsu, 2001 [7]	70, M	Iliopsoas abscess	DRESS syndrome	Oxacillin, metronidazole, ceftriaxone	Acute TIN
5	Sokol et al. 2004 [8]	71, F	Pneumonia	Hypertension	Piperacillin/tazobactam, amikacin	ATN
6	Wicklow et al. 2006 [9]	8, M	VP shunt infection	Obstructive hydrocephalus	Ceftriaxone, cefotaxime, cloxacillin	ATN
7	Wu et al. 2007 [10]	13, M	Skin infection	SLE	None	ATN, LN class V
8	Hong et al. 2007[11]	44, M	Osteomyelitis		Not known	Granulomatous TIN
9	Salazar et al. 2010 [12]	51, M	Osteomyelitis	Rash	Not known	Acute TIN
10	Sha-Khan et al. 2011 [13]	23	IV line infection	Acute leukemia in remission	Piperacillin/tazobactam	ATN
11	Htike et al. 2012 [14]		Bacteremia		Not known	Acute TIN
12	Gelfnad et al. 2014 [15]	45, F	Osteomyelitis	Hypertension, type II DM	None	Acute TIN, moderate DN
13	Gelfnad et al. 2014 [15]	61, M	Wound infection	Hypertension, gout	None	Acute TIN, IgA nephropathy
14	Kim et al. 2016 [16]	11, M	Parotitis	DRESS syndrome	Ceftriaxone	ATN, postinfectious GN
15	Katikaneni et al. 2016 [17]	53, M	Lung abscess		Piperacillin/tazobactam	Acute TIN, ATN
16	Katikaneni et al. 2016 [17]	57, F	Osteomyelitis		Cefepime	ATN
17	Katikaneni et al. 2016 [17]	64, M	Infected knee	Hypertension, type II DM	None	ATN
18	Pingili and Emmanuel, 2017 [18]	79, M	Bacteremia	Leukocytoclastic vasculitis of skin	None	Acute TIN
19	Sawada et al. 2018 [19]	41, M	Genital infection		Piperacillin/tazobactam	Acute TIN, ATN

ATN = acute tubular necrosis; DM = diabetes mellitus; DN = diabetic nephropathy; DRESS = drug reaction with eosinophilia and systemic symptoms; GN = glomerulonephritis; IV = intravenous; LM = light microscopic; SLE = systemic lupus; TIN = tubulointerstitial nephritis; VP = ventriculoperitoneal.
